# Tracking Progress of National Health Summit Declarations - Health Policy Analysis: A Qualitative Study

**DOI:** 10.31729/jnma.8871

**Published:** 2025-01-31

**Authors:** Ashis Shrestha, Lisasha Poudel, Dipesh Tikhatri, Sushant Regmi, Jay Bhushan Jha, Shreya Baniya, Mandish Prasad Phuyal, Akanshya Prasai, Sunil Kunwar, Amit Chand, Prakriti Karki, Nurusha Tamrakar, Ritu Sapkota, Raman Yadav

**Affiliations:** 1 Patan Academy of Health Sciences, Lagankhel, Lalitpur, Nepal; 2 Institute for Implementation Science and Health, Kathmandu, Nepal; 3 Nepal Medical Association, Exhibition Road, Kathmandu, Nepal; 4 Kathmandu Medical College and Teaching Hospital, Sinamanal, Kathmandu, Nepal; 5 Hope International College, Mahalaxmisthan, Lalitpur, Nepal

**Keywords:** *coordination*, *declaration*, *health policy*, *health system, policy implementation*

## Abstract

**Introduction:**

The National Health Summit is a policy discussion platform that advocates the policy statement stated by National Health Policy 2019 which results in a set of declarations to address issues in the health system. This study's objective is to monitor the extent of declaration's implementation, its facilitators-barriers, and to formulate recommendation for future National Health Summit declarations and implementation strategies.

**Methods:**

This is a qualitative study conducted by Nepal Medical Association. in November-December 2024. Ethical approval from Nepal Health Research Council, Ethical Review Board (Reference number: 1069). The study was designed using the Realistic Evaluation Framework to look into the "context" which are the external factors impacting implementation of the declarations, the "mechanism" which are key elements facilitating or hindering policy changes, and the "outcome" which were the tangible results of NHS declarations.

**Results:**

Review and analysis of the participant responses identified policy influene and advocacy as contexts. The mechanisms identified were political and bureaucratic challenges, collaboration, finaicial hurdles, commitment, accountability, leadership, evidence based practices. The outcome discussed were health system strengthening, health workforce, governance, innovation, advocacy and digaital transformations.

**Conclusions:**

Implementation of declaration is challenging, with fragmentation as major health issues, however, continuous advocacy has had a synergistic effect in implementation and prioritization of the policy.

## INTRODUCTION

Nepal is a Democratic country within a Federal Republic where the right to health is guaranteed as a fundamental right for all citizens. The right to health is provided by Section 35 of the constitution of Nepal.^1^ Therefore, this right has been secured in the policy statement. The National Health Summit (NHS) is a policy discussion platform that advocates the policy statement stated by National Health Policy 201 9^2^ (NHP-2019) aligning with the principles, objectives, and outcomes stated by National Health Sector Strategic Plan 2023-2030.^3^ The platform has considered Public Health Service Act 2019^4^ and Public Health Service Regulations as a foundation for the policy discussion.^5^ This is an annual summit that started from 2021 and each summit concludes with declaration that is handed over to the Ministry of Health and Population.

Each summit results in a set of declarations to address important issues in the health system. However, it remains unclear to what extent these declarations have been implemented or how effective they have been in achieving their intended goals. Therefore, this study seeks to answer these questions by conducting qualitative research through Key Informant Interview with government officials, healthcare providers, policymakers, and non-governmental organizations. The aim of this study is to explore the extent to which NHS declarations have been implemented, its facilitators-barriers, and to formulate recommendations to strengthen future National Health Summit declarations and implementation strategies.

## METHODS

This is a qualitative study conducted by Nepal Medical Association (NMA) which is a non-profit national professional organization of Medical and Dental Doctors of Nepal, established in 1951. National Health Summit is a calendar activity of NMA conducted yearly since 2021 with academic support from Journal of Nepal Medical Association (JNMA). The NHS is a policy discussion platform consisting of several panel discussion over two days with participation of stakeholders of health system of Nepal. The declaration of NHS first, second and third included following important issues: pandemic preparedness, multilateral coordination, self-sufficiency in medical supplies, infrastructure, workforce development, health worker safety, equitable access, quality care through innovation, public-private partnerships, brain drain vs brain gain and evidence-based policies.

This study was conducted in November-December 2024, data collection was started after ethical approval from Nepal Health Research Council, Ethical Review Board (Reference number: 1069). The study was designed using the Realistic Evaluation Framework^6^ to look into the "context" which are the external factors impacting implementation of the declarations, such as political support, organizational capacity, and public health needs; the "mechanism" which are key elements facilitating or hindering policy changes, including coordination, finance; and the "outcome" which were the tangible results of NHS declarations.

The study population included politician (1), regulatory bodies (3), government officials (n=2), academy (3), professional societies (6). Individuals without direct knowledge or involvement in NHS-related policy or implementation were excluded from the study. Purposive sampling was done to identify key persons who were involved in or have oversight of the NHS declarations. The sample size was guided by data saturation, which in this study was achieved after conducting 15 interviews.

Key Informant Interviews (KIIs) was conducted to gather in-depth insights from stakeholders involved in the implementation of the NHS declarations.

The participants included: Vice Chairperson - Medical Education Commission, Secretary - Ministry of Health and Population, President- Nepal Medical Council, ViceChancellor - National Academy of Medical Sciences (NAMS), Vice Chancellor- Madan Bhandari Academy of Health Sciences, Rector - National Academy of Medical Sciences (NAMS), Director General - Department of Drug Administration, Director - Curative Service Division, Mayor - Rajpur Municipality, President - Nepal Medical Association (NMA), General Secretary - Nepal Medical Association (NMA), President - Association of Private Health Institution of Nepal, Chairman - Association of Private Medical and Dental Colleges of Nepal, President - Association of Pharmaceutical Producers of Nepal, President - America Nepal Medical Foundation.

A structured interview guide was used to ensure consistent, relevant questioning across participants while allowing for flexibility in responses. To ensure data reliability, the interview guide was pre-tested with two similar study participants to assess its clarity and relevance. The duration of interview was from 30 to 45 minutes and was conducted after written informed consent. The interview was recorded and the conversation was in Nepali Language. A principle investigator oversaw interview sessions, data recording, and had regular checks for data accuracy and adherence to research protocols. Following the interview, the audio record was transcribed and translated into English. Two researcher independently coded data and thematic analysis was done. Themes were discussed to formulate the final consensus themes. The pressing issues and challenges described by the participants on implementation of the policy as advocated by declaration were collected, collated, interlinked. Deductive reasoning was done to identify the major issues and concerns.

## RESULTS

Based on the interview sub themes were derieved on the contexts, mechanism and outcome (Table 1).

**I. Contexts**

Policy Influence and Advocacy: Participants emphasized that the NHS serves as a crucial platform for policy discussions, advocating for healthcare workers' rights, enhancing health policies. They also noted that the platform promotes stakeholder engagement and ensures follow-up on key health issues, increasing its effectiveness over time.

**Table 1:** Key themes and sub-themes.

THEME		SUB-THEME
Context		Policy Influence and Advocacy
Mechansim	Pushers and barriers for NHS declaration	Political and Bureaucratic Challenges
		Collaboration and Stakeholder Engagement
		Financial hurdles
		Commitment, Accountability, and Leadership
		Follow-up Mechanism
	Health System issues that needs to be discussed	Health System Budget
		Research and Evidence-Based Practices		
		Quality and Safety
Outcome		Healthcare System Strengthening and Service Expansion
		Health Workforce
		Policy Development and Governance
		Innovation, Advocacy, and Digital Transformation

"The NMA has been at the forefront of addressing health sector challenges, including policy discussions, implementation issues, and political movements. It has consistently pushed for improvements in healthcare, using the National Health Summit (NHS) as a platform to engage various stakeholders, including the government, healthcare professionals, and other relevant sectors."

"This event has created opportunities for better collaboration, influencing local and national policy regarding public health priorities and setting a direction for future health initiatives."

**II. Mechanisms**

**1. Pushers and barriers for NHS declaration**

Participants discussed factors influencing the implementation of declarations, identifying both enablers and obstacles.

**a. Political and Bureaucratic Challenges**

Participants stressed that politics heavily influences the medical profession, shaping healthcare decisions and creating barriers to progress. They further noted that bureaucratic challenges and frequent changes in leadership disrupt the effective implementation of declarations. Advocacy initiatives, they emphasized, are often weakened by political biases and a lack of unified accountability.

"The biggest challenge, in my opinion, is political commitment. Our health has always been a part of the election agenda for political parties, but when it comes to implementation, it is often overlooked."

"Now, obviously, the primary responsibility for health lies with the Ministry of Health, but the political changes within the ministry have been significant. If you observe, within these three years, it's possible that nine or ten ministers might have changed."

**b. Collaboration and Stakeholder Engagement**

Participants identified collaboration and stakeholder engagement as key factors for the successful implementation of declarations. They emphasized that addressing health issues requires a multisectoral approach, involving bureaucrats, politicians, civil society, and other stakeholders. Declarations should align with diverse viewpoints and be grounded in common consensus. Additionally, they noted that plans must incorporate collaboration, adequate budgeting, and the inclusion of stakeholders at every level.

"When we come to the third point, health must go beyond because health is related to a multistakeholder approach... and health is associated with the Ministry of Health. Why? Because this is a shared responsibility, and when everyone contributes, improvement can be seen. If we focus solely on one ministry, it becomes a [pause] misconception. A major misconception in our policy is this."

"Health sector needs coordination between beurocrates, politicians. The changes in health is possible only if we move ahead with stakeholders who are not in health sector. Moreover, we should also see the feasibility issues, the agendas that are not feasible at present will take longer duration."

**c. Financial hurdles**

One of the major barriers to effective implementation of declaration as identified by participants were finance related issues. Participants pointed out the absence of adequate budget allocation for implementing the declaration. As participants noted, the Ministry of Finance should allocate sufficient funds, and the Ministry of Health and Population should be empowered to use those resources effectively for procurement and human resource development. However, this balance has yet to be fully achieved, leading to a bottleneck in translating declarations into tangible outcomes.

"For instance, understanding is important because sometimes finance gets involved. When you make a declaration about something, it leads to recommendations, but those recommendations must be implemented. The implementing authority often faces challenges, and due to budget constraints, things don't move forward"

"At present, it seems that there is a lack of coordination between the Ministry of Health and the Ministry of Finance, or perhaps health is not a priority for the Ministry of Finance, or we have not been able to create the necessary level of pressure. The fundamental issue here is political commitment. When we declare health and education as constitutional rights, the question arises as to why the state is not fully implementing them. For instance, if the taxes collected from health-related products were entirely mobilized for the health sector, their priorities would shift significantly."

**d. Commitment, Accountability, and Leadership**

Participants identified a lack of commitment, accountability, and leadership as key barriers. They highlighted that there is a lack of shared responsibility and a failure to understand that the concerns raised are for the collective good, not against any individual. Frequent changes in leadership were also mentioned as a significant challenge to effective implementation.

"Things are not done collaboratively. Another issue is that when it comes to creating the foundation for these declarations, there isn't enough preparation or commitment put into it. The way we approach this often feels like it has been done in an academic or intellectual manner, merely for exploration, rather than in a practical way. These are the main issues I see in this process."

"That's why we at NMA thought that health alone cannot address these issues; every stakeholder must take equal responsibility for improvements in our health system. [Pause] To bring about improvements in health, there needs to be a good sense of discipline, and there must also be integrity."

"Leadership is essential for change. To transform a country, it starts with one person. For example, China, Russia, and Singapore were all shaped by strong leadership. Even a small country like Nepal needs strong leadership with a clear vision to bring about change. Leadership doesn't necessarily mean the prime minister; it applies to every institution. The right leaders should be in the Medical Education Commission, Medical Council, and Ministry of Health. Leadership requires knowledge and vision. But leadership alone isn't enough —it needs a team. For example, in an institution, the leader needs the right people, who are not influenced by politics. They must be the right fit for the role and have the necessary resources to work effectively."

**e. Follow-up Mechanism**

Participants identified the establishment of a robust follow-up mechanism as a key driver for the effective implementation of declarations. They emphasized that simply making recommendations or drafting policies is not sufficient; consistent and structured follow-up with relevant stakeholders, organizations, and individuals is essential to ensure progress, address barriers, and translate declarations into tangible outcomes.

"Continuous follow-up is crucial. For instance, after making a recommendation, it's not enough to just make it and move on; follow-up is needed. There should be continuous communication and coordination with the concerned stakeholders, organizations, and individuals to ensure effective implementation."

"Nepal Medial Association needs to form Research and Development unit and followup the issues in form of policy researches."

**2. Health System issues that needs to be discussed**

**a. Quality and Safety**

Participants raised concerns about the quality of healthcare services and the safety within the healthcare system. A key issue identified was the violence against healthcare workers, which they emphasized requires urgent and comprehensive discussions to find effective solutions. Additionally, some participants stressed the importance of implementing Minimum Service Standards (MSS) as a critical step toward ensuring consistent, high-quality healthcare services.

"MSS has done a good job in uplifting the standards of our hospitals, I think this should also be implemented in hospitals."

**b. Research and Evidence-Based Practices**

Participants emphasized the critical importance of fostering evidence-based practices within the healthcare system. They underscored the need for research findings to inform policy and practice, highlighting a significant gap in the translation of evidence into actionable strategies. Despite a growing body of research, participants noted that its integration into decision-making processes remains inconsistent, costly, limiting the potential for improved health outcomes.

"Research is currently underway, but the critical question is how to translate this research evidence into actionable policy or practice. This is not just a local issue but a global challenge. The key message here is the importance of trusting in science. Scientific research generates credible evidence that can drive positive change, but we face difficulties in conveying this evidence clearly and effectively to policymakers. During this summit, while there has been input from external participants, I feel that their comments have not had a significant impact. More importantly, there seems to be a lack of proper follow-up on the actions or recommendations discussed, which undermines the effectiveness of the entire process."

"At present we are conducting product statility researches, researches for making new drugs is costly. It will cost about 3-10 billion dollar for 20 years to make one new drug. These are beyond our capacity, therefore it is essential to collaborate."

**c. Health System Budget**

A pressing issue identified by participants was the urgent need to increase budget allocation for the health system. Participants repeatedly emphasized that the current financial support for healthcare is insufficient to meet growing demands and address critical gaps in infrastructure, human resources, and service delivery. The lack of dedicated funding for essential healthcare services undermines efforts to improve accessibility, quality, and equity across the system. Moreover, participants pointed out the disconnect between policy aspirations and budgetary provisions, which hampers effective implementation of healthcare initiatives.

"The Nepal government has not been able to allocate even 10% of the budget for the health sector. I think in other sectors, the budget system in Nepal is more visible, but in health, we can see only 4 to 5% allocated. As a result, government hospitals, primary health centers (PHCs), and health posts are facing a situation where the services that could be provided are limited." - KII05

**III. Outcome (Accomplishment of NHS)**

**a. Healthcare System Strengthening and Service Expansion**

Participants highlighted key accomplishments in strengthening the healthcare system and expanding services following the National Health Summit. They emphasized the establishment of a dedicated mental health section within the Department of Health Services. Additionally, respondents highlighted the ongoing expansion of healthcare services at the local level.

"We have already established a separate structure for mental health.... Similarly, previously mental health and NCDs were together, but now we have also established a separate structure for NCDs."

A participant emphasized the critical importance of exploring home-based care implementation, highlighting that the NHS played a key role in sparking these topic discussions, with its declaration providing a platform for the broader acceptance and integration of home-based care into the healthcare system.

"We organized an event to explore whether home-based care can be implemented. In this, we are making efforts, practicing, and also considering how successful we can be in this area. The declaration made during the National Health Summit, also appears to have opened a path for this, as I see it."

One participant highlighted how the National Health Summit has facilitated the expansion of services, including the establishment of oxygen plants, infectious disease units in various hospitals, and efforts to combat antimicrobial resistance.

"If we look at it now, there have been a few infectious units and hospitals established in Nepal, haven't there?"

**b. Health Workforce**

Respondents emphasized achievements in human resources in health, including capacity building and education. They noted that staffing challenges are being addressed through Organization and Management (O&M) efforts at both federal and local levels. One participant highlighted the implementation of "Brain Gain" initiatives aimed at facilitating the return of skilled professionals to the country for longterm contributions.

"I am also trying to influence this area, especially when it comes to leveraging brain gain. We are doing our best. We want to make it easier for those coming from outside. In the past, it was very difficult for them to register. But now, registration has become much easier for us. As a result, when someone comes here and says they want to work at an institution or in academia, there is no longer a problem".

Others pointed out the transformative impact of the NHS declaration on medical education, including the introduction of additional seats for medical education. Participants also emphasized the collaborative mapping of healthcare workforce needs by the Medical Education Commission and the Ministry of Health and Population.

"Last time we discussed medical education, and this time, the Nepal government has granted MBBS seats to three medical colleges."

**c. Policy Development and Governance**

Participants emphasized key accomplishments in policy development and governance. They noted ongoing efforts to address violence within the healthcare system, alongside initiatives aimed at rebuilding the federal health structure and establishing a resilient healthcare system.

"These issues that we raised at the National Health Summit, particularly the resilient health system we started focusing on during the COVID period, have definitely seen some positive beginnings over these three years as we now approach the fourth summit."

One participant mentioned budget allocation for the preparation of policy guideline focused on health worker safety.

"We are considering creating a set of guidelines, and in this year's plan, we have allocated some budget. The focus is on ensuring safety in the workplace for doctors, and we are progressing with this process. We are exploring what can be done to improve safety in their working environment." - KII05

**d. Innovation, Advocacy, and Digital Transformation**

Participants emphasized the role of innovative initiatives being advocated following the National Health Summit. One participant noted that the NHS has supported journalism as a tool for advocacy and awareness-raising while encouraging the adoption of digital health solutions to improve healthcare delivery and access.

"Now we have discussed digital health, and currently, there is a digital health policy in place, so things have been moving forward".

"Similarly, last year we started discussing health journalism, and now there is a trend in mainstream media to include the voices of stakeholders when covering health-related topics."

Participants stated that the implementation of the declaration is a long term process and short term gain may not be seen, we need to keep on going.

"We will do advocacy, you will not listen, but still we will keep on doing advocacy."

"This is a long term process, in fact you cannot meaure it in terms of gain or loss, this is a process which needs to keep on going."

**Synthesizing Themes**

Based on the interviews, the major problem identified was in coordination. Combination of various factors leading to fragmented health care was observed to be a catalyst for the incoordination.

"We demonstrate reactivism, interprofessional relationship are for formality only."

"When I was working as an official of provincial government, it was difficult for me to get work done at hospital run by local government."

"The local government are not given full authority to work independently, there are hurdles from various level of government."

**Figure 1:**
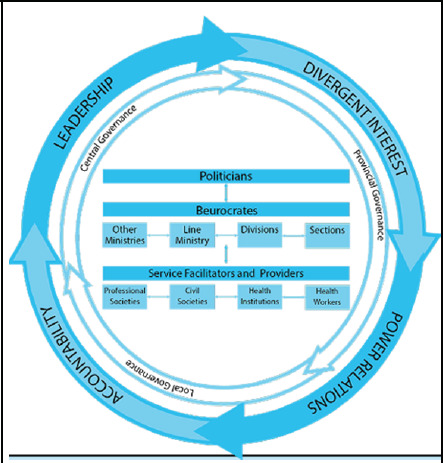
Responsible factors for fragmented health care.

The key factors identified for fragmented health care are: Human factors, which consists of professionals related to health service providers/receivers, bureaucrats, and politicians; system factors, which include local, provincial, and central governance; and cross-cutting factors which include leadership, divergent interest, power relation and accountability (Figure 1).

## DISCUSSION

The research findings stated that the National Health Summit is an important policy discussion platform and a calendar event of the Nepal Medical Association. Unlike other scientific events that foster knowledge and networking, this event also provides the same opportunity.^7^ However, as all scientific contents are in the form of panel discussions, this is also a platform for brainstorming, creating awareness, and disseminating policy statements.

The participant reported that the declaration of the NHS has not been adequately communicated to the necessary stakeholders. This probably has three dimensions. The first is the unavailability of a follow-up mechanism at the Nepal Medical Association. The action plan needs to be clear, with a follow-up plan and commitment. Furthermore, the interviews also recommended a research and development unit so that policy dialogue and advocacy are followed up, documented, researched, and published. Secondly, something that is good for one might not be good for another. Based on this, the implementation policy is defined in four paradigms: low-conflict-low-ambiguity (requires administrative implementation), high-conflict-low-ambiguity (requires political implementation), high-conflict-high-ambiguity (requires symbolic implementation), and low-conflict-high-ambiguity (requires experimental implementation).^8^

Finally, the third is due to the multi-layered process of policy implementation in Nepal. In order to implement policy in Nepal, the National Planning Commission must establish the national development goal based on the policy, its declaration, input from past periodic plans, international commitments, public needs analysis, development indicator evaluation, and resource availability.^9^ The entities involved in the planning are the National Development Council, National Planning Commission, Line Ministries, Ministry of Finance, and Cabinet.^10^ Therefore, this is a time-consuming process requiring perseverance and persistence. Vanni Agnoletti et al. stated that the healthcare system is an infinite game.^11^ In contrast to finite games, which have rules, winners, and losers, infinite games have no winners or losers, are never-ending, and have rules that must inevitably change while the game is being played. The objective of the infinite game is to bring as many people as possible into the play.^12^ A finite strategy in an infinite process will lead to failure. Thus, the implementation of the NHS declaration is an infinite process and will contribute to policy shaping bit by bit. Hence, it is important to keep advocating. The implementation is, therefore, multifactorial and multidimensional, so it might be beyond the scope of the NHS. However, the follow-up mechanism will ensure that the issues are given priority.

Despite the challenges, the National Health Summit declaration has helped in leveraging some policy implementations. The establishment of separate frameworks for mental health (NHP-2019, Section 6.17) and non-communicable diseases (NCDs) (NHP-2019, Section 6.12) is one notable achievement. Capacity-building efforts were yet another landmark achievement. Critical care training and the installation of oxygen plants have enhanced the resilience of Nepal's health system, particularly during crises like the COVID-19 pandemic (NHP-2019, Section 6.11). Transforming medical education has also been a priority, with initiatives to align educational outputs with the country's healthcare needs. The Medical Education Commission has been undertaking faculty mapping, which is essential for improving medical education opportunities in the country. The interview also stated that there has been an effort to map human resources in the country by the Ministry of Health and Population, which will help create job opportunities (NHP-2019, Section 6.8). Furthermore, advocacy and awareness initiatives have strengthened the NHS's impact by addressing critical issues like antimicrobial resistance (NHP-2019, Section 6.24), digital health (NHP-2019, Section 6.15), and healthcare worker safety as per the ordinance on the safety and security of health workers.^13^

At present, the greatest challenge faced by the healthcare system is coordination. Fragmentation is the major frictional force for suboptimal coordination in the healthcare system.^14^ Globally, four identified causes of fragmentation are: problems of global leadership, divergent interests, problems of power relations, and problems of accountability.^15^ Furthermore, the federal, provincial, and local governments work together but face challenges in coordination. To manage the connection between the three levels, intergovernmental structures like the Provincial Coordination Council and Inter-Provincial Council have been formed.^16^ There is a complex problem of coordination between and within three entities, which are: healthcare workers, professional societies, and civil societies; bureaucratic mechanisms that include the line ministry, divisions, sections, and other ministries; and political bodies with various ideologies.

At the ground level, the situation is made worse by limited financial allocation for health, workforce limitations such as unequal pay, insufficient resources, and occupational risks, which lower morale and productivity. Almost all participants raised the concern of violence against healthcare professionals as a demotivating factor. The growing expectations of the public, resource limitations in providing quality care services, and a disconnect between the public and healthcare workers are major factors contributing to the increase in violence. Overcoming these challenges requires versatile and efficient leadership. Therefore, leadership is a vital element of health system development and reform. As Frenk has argued: "Probably the most complex challenge in health systems is to nurture persons who can develop the strategic vision, technical knowledge, political skills, and ethical orientation to lead the complex processes of policy formulation and implementation. Without leaders, even the best-designed systems will fail."^17^

Leadership in this everyday politics is central to health policy implementation. Nurturing these new forms of political leadership within health systems will, however, require new approaches to leadership development. No single lead actor, institution, or process exists that can harmonize the problems. Nevertheless, leadership development should adopt an apprenticeship approach, such as including leadership in the curriculum of medical education at all levels, offering workplace-based lifelong learning, ensuring effective leadership, and advancing health system improvements.^18^

The experts and stakeholders suggested that the discussion should continue to advocate for practice-changing and collaborative research (NHP-2019, Section 6.14), human resources (NHP-2019, Section 6.8), health financing (NHP-2019, Section 6.21, 8), quality and equitable healthcare (NHP-2019, Section 18), a one-door and flexible insurance system (NHP-2019, Section 6.2), brain drain and brain gain, a robust medical education system (NHP-2019, Section 6.6), protection of healthcare worker rights, and measures to nullify violence against healthcare workers.

The study includes an Key Informant Interview of all stakeholders (healthcare workers and bureaucrats) at all three layers of the healthcare system. The implementation of the policy as advocated by declaration was explored however, political representation was limited to the local level. Furthermore, more representation in all category from the provincial and central levels would have added to the perspectives in the findings.

## CONCLUSIONS

National health summit is an essential platform for policy discussion. Implementation of declaration is challenging as it is multi-factorial however, continuous advocacy is necessary to make changes. The advocacy has had a synergistic effect in implementation and prioritization of the policy. The study identified fragmentation as major health issues that needs to be addressed to improve the coordination amongst various stakeholders.
